# Preliminary Application of Magnetization Transfer Imaging in the Study of Normal Uterus and Uterine Lesions

**DOI:** 10.3389/fonc.2022.853815

**Published:** 2022-07-14

**Authors:** Qiu Bi, Qing Li, Jing Yang, Junyu Yang, Ji Du, Fan Ding, Yunzhu Wu, Shaoyu Wang, Ying Zhao

**Affiliations:** ^1^ Department of MRI, The First People’s Hospital of Yunnan Province, The Affiliated Hospital of Kunming University of Science and Technology, Kunming, China; ^2^ MR Scientific Marketing, Siemens Healthineers, Shanghai, China

**Keywords:** magnetization transfer, MRI, normal uterus, uterine lesions, cervical cancer, endometrial cancer

## Abstract

**Purpose:**

The aim of this study is to evaluate the utility of magnetization transfer (MT) imaging in the study of normal uterus and common uterine lesions.

**Methods:**

This prospective study enrolled 160 consecutive patients with suspected uterine lesions. MT ratio (MTR) map was obtained by pelvic MT imaging on a 3.0T MRI scanner. Patients confirmed by pathology were divided into microscopic lesion group and lesion group, according to whether the maximum diameter of the lesion was less than 5 mm. After evaluating and eliminating patients with poor image quality by a three-point Likert scale, MTR values of lesions and normal endometrium, myometrium, and cervix were independently measured on the MTR map by two radiologists. Inter-reader agreement was evaluated. MTR values were compared among different uterine lesions and normal uterine structures using the Mann–Whitney U test with Bonferroni correction. Receiver operating characteristic curve was performed. The correlations between age and MTR values were explored by Pearson correlation analyses.

**Results:**

A total of 96 patients with 121 uterine lesions in the lesion group and 41 patients in the microscopic lesion group were measured. The MTR values among normal endometrium, myometrium, and cervix were statistical significant differences (*P* < 0.05). There were significant differences between endometrial cancer and normal endometrium and between cervical cancer and normal cervix (both *P* ≤ 0.001). Area under the curve (AUC) for diagnosing endometrial and cervical cancer were 0.73 and 0.86. Myometrial lesions had significantly higher MTR values than endometrial lesions and cervical cancer (both *P* < 0.001), and the AUC for differentiating myometrial lesions from them were 0.89 and 0.94. MTR values of endometrial cancer were significantly higher than those of cervical cancer (*P* = 0.02). There was a critical correlation between age and MTR values in endometrial cancer (*r* = 0.81, *P* = 0.04).

**Conclusions:**

MTR values showed significant differences among normal uterine structures. It was valuable for diagnosing and differentiating uterine cancer. MTR values could differentiate myometrial lesions from endometrial or cervical lesions.

## Introduction

Common uterine lesions include endometrial cancer, cervical cancer, and leiomyoma. According to Globocan 2018 estimates, endometrial cancer and cervical cancer are the most common malignant uterine tumors in developed and developing countries, respectively, and rank sixth and fourth in the world for their incidence rates, respectively (1). Surgery is the most important way to treat endometrial cancer (2). Management of cervical cancer is stage-specific and involves chemoradiotherapy (3). Uterine leiomyoma is the most common benign uterine tumor and can be treated with nonsurgical options (4). Therefore, it is essential to determine the origins of the uterine lesions prior to treatment as management strategies differ.

MRI is currently a common imaging method for non-invasive detection and evaluation of uterine lesions (5, 6). In particular, it is valuable for the differentiation of benign and malignant uterine diseases and preoperative staging of malignant tumors (7, 8). Conventional T2-weighted imaging (T2WI) and some functional MRI sequences such as diffusion-weighted imaging (DWI) and dynamic contrast-enhanced MRI (DCE-MRI) have been widely explored for diagnosing uterine diseases (9). However, because of coexisting multiple lesions, extensive lesions, metratrophia, and other factors, the accuracy of MRI in identifying different primary uterine lesions needs further improvement, especially for cancers involving both cervix and the lower uterine segment, leading to ambiguous diagnosis of endometrial and cervical cancer (10). Because both of them showed high signal on T2WI, obvious high signal on DWI, and mild enhancement on contrast-enhanced MRI (CE-MRI) (11). Novel imaging techniques that could reveal histological origins of uterine lesions are needed in clinical practice.

Magnetization transfer (MT) imaging can indirectly reflect the content of structural macromolecular substances (such as protein, lipid, and nucleic acid) in biological tissues by quantitatively measuring MT ratio (MTR) values (12). This parameter represents the efficiency of the magnetization exchange between the protons bound to macromolecules and the relatively free water protons inside tissue (13). Any pathological change in cell macromolecules will cause a change of MTR value. This technique has already been well applied in the study of glioma histological grade (14), assessment and identification of brain tumors (12, 15, 16), and evaluation of intestinal fibrosis in Crohn’s disease (17, 18). However, the value of MT imaging in the uterus was uncertain. The tissue compositions of different structures of normal uterus and uterine lesions of different histological origin are various. We speculate that their contents of macromolecular substances may be different; hence, the MTR values may be different. As a consequence, the purpose of this study was to preliminarily evaluate the value of MT imaging in the study of normal uterine structures and common uterine lesions and to explore the correlations between age and MTR values of the different uterine structures or different uterine lesions.

## Material and methods

### Study Population

**Figure 1:**
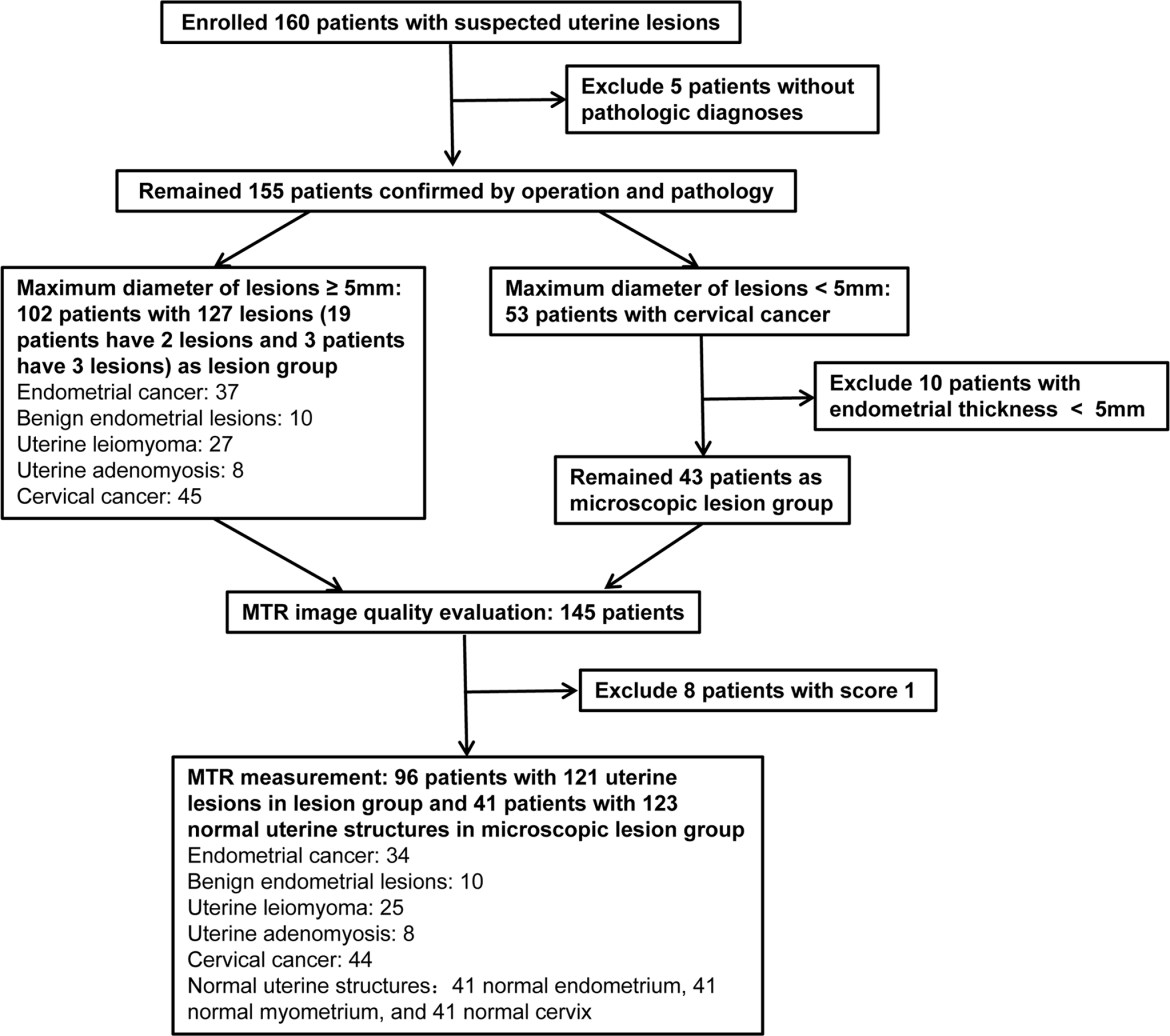
Flowchart of the study.

This prospective study has been approved by our hospital ethics committee and the informed consent of all patients. A total of 160 consecutive patients with suspected uterine lesions were recruited from January 2021 to November 2021. All patients underwent routine MRI and MT imaging scanning. Five patients who did not have a pathological diagnosis were excluded. The remaining 155 patients received operation and pathological examination after MR scanning within 2 weeks. According to whether the maximum diameter of the lesion was less than 5 mm, the patients were divided into microscopic lesion group and lesion group. The lesions of the microscopic lesion group were virtually detected only by microscopy. Because we need to measure MTR values of normal endometrium in the microscopic lesion group, 10 patients with endometrial thickness less than 5 mm were excluded. Finally, only 43 patients were included in the microscopic lesion group. The study population flowchart was presented in **Figure 1**.

### MRI Protocol

Pelvic MRI scanning was performed on a 3.0T MRI scanner (Magnetom Prisma, Siemens Healthineers, Erlangen, Germany) with an eight-channel phased-array abdominal coil. All patients were told to abstain from food and drink for at least 4 h before MRI examination. To reduce the air in the rectum and sigmoid, patients were prepared with 10 ml of glycerin enema administration into the rectum 30 min before MR scanning. All patients were scanned in a supine, feet-first position with a properly inflated bladder. The routine MR protocols included T1-weighted imaging (T1WI), T2WI, DWI, and DCE-MRI. Uterus-axial DWI was performed using ZOOMit techniques based on echo planar imaging combined with reduced volume excitation by setting standard b value of 50 and 1,000 s/mm^2^. Sagittal DCE-MRI was performed using three-dimensional volumetric interpolated breath-hold examination sequence by continuous scanning at 10 stages immediately after intravenous injection of contrast agent. The late CE-MRI included axial, sagittal, coronal, and uterus-axial scanning. The contrast agent that we used was gadolinium meglumine (0.2 ml/kg), intravenously injected at a rate of 1.5 ml/s, and then washed with 10 ml of saline at a rate of 2 ml/s. A two-dimensional fast low-angle shot sequence was used to acquire MT imaging data before enhanced scanning, including two scan with (MT_on_) and without (MT_off_) MT pulse, respectively. The total imaging time of MT imaging was 2 min 42 s. For MT quantification, the MTR map was calculated on the MR scanner workstation using the following formula: MTR = (MT_off_ − MT_on_) × 100/MT_off_. The routine details of scanning parameters were shown in **Table 1**. More parameters of MT imaging were as follows: saturation pulse, Gaussian radio frequency (RF) pulse; amplitude, 375 Hz; length, 9.984 ms; and off-resonance frequency, 1.2 kHz.

**Table 1:** MR imaging parameter details.

Sequences	Repetition Time (ms)	Echo Time (ms)	Field of View (mm^2^)	Matrix	Slice Thickness (mm)	Slice Gap (mm)	Flip Angle
Uterus-axial MTI	222	2.35	380 × 380	256 × 208	4	4.8	70°
Axial T2WI	8230	97	360 × 360	384 × 384	5	6	120°
Sagittal T2WI	5470	89	210 × 210	384 × 384	3	3.6	130°
Coronal T2WI	4000	78	360 × 360	384 × 384	4	4.4	150°
Uterus-axial T2WI	3200	90	200 × 200	320 × 320	3	3.6	130°
Axial T1WI	480	10	346 × 313	384 × 384	5	6	120°
Uterus-axial DWI	6300	75	250 × 134	72 × 134	3	3.6	90°
Axial CE-T1WI	2.9	1.09	346 × 313	290 × 320	3	0	12.5°
Sagittal CE-T1WI	3.92	1.46	260 × 260	320 × 320	3	0	9°
Coronal CE-T1WI	3.92	1.39	300 × 300	320 × 320	3	0	9°
Uterus-axial CE-T1WI	2.9	1.19	220 × 200	288 × 262	3	0	12.5°

MTI, magnetization transfer imaging; T2WI, T2-weighted imaging; T1WI, T1-weighted imaging; DWI, diffusion weighted imaging; CE, contrast-enhanced.

### Image Quality Evaluation and Measurement

**Figure 2:**
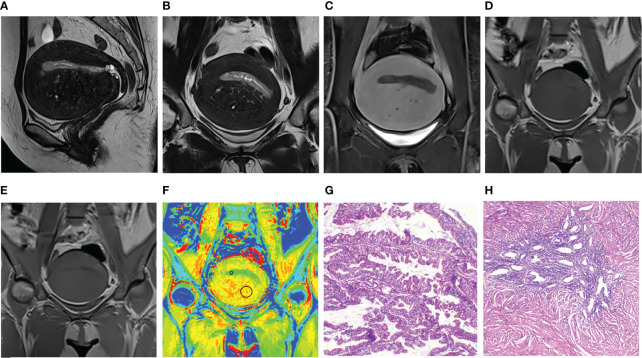
Images and placements of the regions of interest in a 48-year-old patient with endometrial cancer and adenomyosis. **(A–C)** Sagittal and uterus-axial T2-weighted images (T2WI) and uterus-axial contrast-enhanced T1-weighted (T1WI) image. **(D, E)** Uterus-axial magnetization transfer (MT) imaging with and without MT pulse. **(F)** Pseudo-color magnetization transfer ratio (MTR) map; MTR values of endometrial cancer and adenomyosis are 8.07 and 12.31, respectively. **(G, H)** Hematoxylin and eosin staining map (40×) of endometrial cancer and adenomyosis.

All MTR maps were transferred to a workstation (Syngo.via Client 4.2) for measurements. One radiologist with 25 years of experience in diagnosing gynecological MR images reviewed and evaluated all the MTR maps’ quality by a three-point Likert-scale: score 1, poor image quality with obvious artifacts, the lesions cannot be detected or distinguished from surrounding structure; score 2, good image quality with few artifacts, the lesions can be identified by reference to other MR images; and score 3, excellent image quality without artifacts, the lesions can be easily detected on MTR maps. Two readers with 6 years of experience in pelvic MRI independently measured MTR values on MTR maps in patients with good and excellent image quality (score 2 and score 3). Referring to other routine MR images, a rounded sizeable region of interest (ROI) was drawn on the maximum area of the lesion (lesion group) or of the normal uterine structures including myometrium, endometrium, and cervix (microscopic lesion group). The mean MTR values were recorded. For myometrium, ROIs were drawn covering the junctional zone and outer myometrium. For cervix, ROIs were drawn covering the cervical stroma and muscularis. Inter-reader agreement was evaluated. The placements of ROIs showed in **Figure 2**.

### Statistical Analysis

Statistical analyses were performed with SPSS software, version 26.0 (SPSS, Inc., Chicago, IL) for Windows. As continuous variable, MTR value was expressed as arithmetic means and standard deviation. Inter-reader agreement was evaluated using the intraclass correlation coefficient. The Shapiro–Wilk test or Kolmogorov–Smirnov test was used to test the normality of the data distribution. The data in each group were not normally distributed, and non-parametric test was performed. The Kruskal–Wallis H test was used to compare MTR values among the three groups with a value of *P* < 0.05. The Mann–Whitney U test with Bonferroni correction was further used for pairwise comparisons, and the adjusted significant level was 0.017 (0.05/3). Receiver operating characteristic curves were performed to diagnose or distinguish the uterine diseases and to determine the optimal threshold values. Pearson correlation analyses were performed to evaluate the correlations between age and the MTR values. A *P*-value less than 0.05 was considered to be correlated. A value of *r* > 0 indicates a positive correlation between the two variables; otherwise, a negative correlation exists.

## Results

MTR image quality of 102 patients with 127 uterine lesions (37 lesions of endometrial cancer, 10 lesions of benign endometrial lesions, 27 lesions of leiomyoma, eight lesions of adenomyosis, and 45 lesions of cervical cancer) in the lesion group and 43 patients in the microscopic lesion group were evaluated. In lesion group, 19 patients have two lesions and three patients have three lesions. MTR image quality scores were summarized in **Table 2**. Fifteen lesions in the lesion group and three patients in the microscopic lesion group exhibited excellent image quality (score 3), and 106 lesions in the lesion group and 38 patients in the microscopic lesion group showed good image quality (score 2). Three lesions of endometrial cancer, two lesions of leiomyoma, one lesion of cervical cancer, and two patients in the microscopic lesion group were excluded with poor image quality due to artifacts. Finally, a total of 96 patients with 121 uterine lesions (34 lesions of endometrial cancer, 10 lesions of benign endometrial lesions, 25 lesions of leiomyoma, eight lesions of adenomyosis, and 44 lesions of cervical cancer) in the lesion group and 41 patients with 123 normal uterine structures (41 normal endometrium, 41 normal myometrium, and 41 normal cervix) in the microscopic lesion group were measured. Interobserver agreement for the measurement of MTR values was presented in **Table 3**. The data of all lesions and structures measured by the two observers had a good consistency. We randomly selected MTR values measured by one of the observers as the final evaluation indices.

**Table 2:** MTR image quality evaluation.

	Score 3	Score 2	Score 1
Endometrial cancer (n = 37)	4	30	3
Benign endometrial lesions (n = 10)	3	7	0
Uterine leiomyoma (n = 27)	5	20	2
Uterine adenomyosis (n = 8)	0	8	0
Cervical cancer (n = 45)	3	41	1
Microscopic lesion group (n = 43)	3	38	2

MTR, magnetization transfer ratio.

**Table 3:** Interobserver agreement for the measurement of MTR values.

	ICC	95% CI
Endometrial cancer (n = 34)	0.87	0.77–0.93
Benign endometrial lesions (n = 10)	0.88	0.73–0.96
Uterine leiomyoma (n = 25)	0.93	0.88–0.97
Uterine adenomyosis (n = 8)	0.89	0.37–0.99
Cervical cancer (n = 44)	0.85	0.59–0.93
Normal endometrium (n = 41)	0.94	0.88–0.97
Normal myometrium (n = 41)	0.98	0.94–0.99
Normal cervix (n = 41)	0.94	0.84–0.98

MTR, magnetization transfer ratio; ICC, intraclass correlation coefficient; CI, confidence interval.

**Figure 3:**
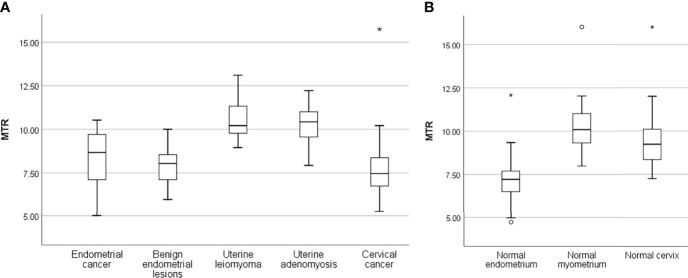
Box plots show the distribution of MTR in uterine lesions **(A)** and normal uterine structures **(B)**. The lines of box plot present the minimum, the lower quartile, the median, the upper quartile, and the maximum, respectively; the length of whiskers and the position of the line in the box suggest the distribution of sample; circle, outliers; *, extremes.

**Figure 4:**
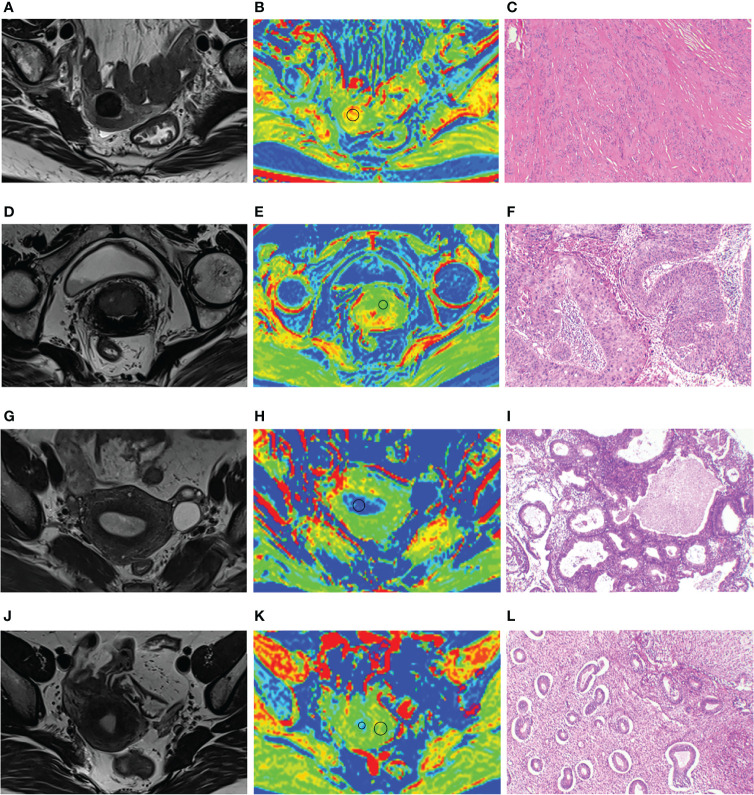
**(A–C)** A 64-year-old patient with uterine leiomyoma, MTR of leiomyoma is 13.01. **(D–F)** A 65-year-old patient with cervical cancer; MTR value of cervical cancer is 7.46. **(G–I)** A 49-year-old patient with endometrial hyperplasia; MTR value of endometrial hyperplasia is 6.93. **(J–L)** A 40-year-old patient with carcinoma *in situ* of cervix; MTR values of normal endometrium and myometrium are 7.17 and 12.03. Panels **(A, D, G, J)** represent uterus-axial T2WIs. Panels **(B, E, H, K)** represent uterus-axial pseudo-color MTR maps. Panels **(C, F, I, L)** represent hematoxylin and eosin staining map (40×) of the lesions.

MTR values in different lesions and normal uterine structures were shown in **Table 4** and **Figures 2**–**4**. MTR values among normal endometrium (7.14 ± 0.21), myometrium (10.18 ± 0.22), and cervix (9.51 ± 0.23) were statistically significant differences (*P* < 0.05). MTR values of normal endometrium were significantly lower than those of normal myometrium and normal cervix (both *P* < 0.001). In addition, MTR values of normal myometrium were significantly higher than those of normal cervix (*P =* 0.008). There was no significant difference among proliferative phase (7.31 ± 0.35), secretory phase (7.16 ± 0.54), and senile endometrium (7.04 ± 0.26) (*P* = 0.89) or among normal myometrium, leiomyoma (10.54 ± 0.23), and adenomyosis (10.27 ± 0.47) (*P* = 0.48). There were significant differences between endometrial cancer (8.29 ± 0.26) and normal endometrium (*P* = 0.001) and between cervical cancer (7.71 ± 0.25) and normal cervix (*P* ≤ 0.001). Myometrial lesions (10.47 ± 1.18) had significantly higher MTR values than endometrial lesions (8.22 ± 1.46) and cervical cancer (both *P* < 0.001). MTR values of endometrial cancer were significantly higher than those of cervical cancer (*P* = 0.02).

**Table 4:** Comparison of MTR values among different groups.

	MTR	*P*	*P*1	*P*2	*P*3
Endometrium group:		0.002*	0.001*	0.06	0.45
Normal endometrium (n = 41)	7.14 ± 0.21				
Endometrial cancer (n = 34)	8.29 ± 0.26				
Benign endometrial lesions (n = 10)	7.99 ± 0.39				
Myometrium group:		0.48	—	—	—
Normal myometrium (n = 41)	10.18 ± 0.22				
Uterine leiomyoma (n = 25)	10.54 ± 0.23				
Uterine adenomyosis (n = 8)	10.27 ± 0.47				
Cervix group:		<0.001*	—	—	—
Normal cervix (n = 41)	9.51 ± 0.23				
Cervical cancer (n = 44)	7.71 ± 0.25				
Uterine lesions of different origin:		<0.001*	<0.001*	0.02*	<0.001*
Endometrial lesions (n = 44)	8.22 ± 1.46				
Myometrial lesions (n = 33)	10.47 ± 1.18				
Cervical cancer (n = 44)	7.71 ± 0.25				
Uterine cancers:		0.02*	—	—	—
Endometrial cancer (n = 34)	8.29 ± 0.26				
Cervical cancer (n = 44)	7.71 ± 0.25				
Normal uterine structures:		<0.001*	<0.001*	<0.001*	0.008*
Normal endometrium (n = 41)	7.14 ± 0.21				
Normal myometrium (n = 41)	10.18 ± 0.22				
Normal cervix (n = 41)	9.51 ± 0.23				
Normal endometrium:					
Proliferative phase (n = 9)	7.31 ± 0.35				
Secretory phase (n = 12)	7.16 ± 0.54	0.89	—	—	—
Senile endometrium (n = 20)	7.04 ± 0.26				

MTR, magnetization transfer ratio; P, comparison among three groups or between two groups with a value of P < 0.05; P1, comparison between the first disease or structure and the second that in each group; P2, comparison between the first disease or structure and the third that in each group; P3, comparison between the second disease or structure and the third that in each group; P1–P3, all using an adjusted significant level, a’ = 0.017.

*, statistically significant difference.

**Figure 5:**
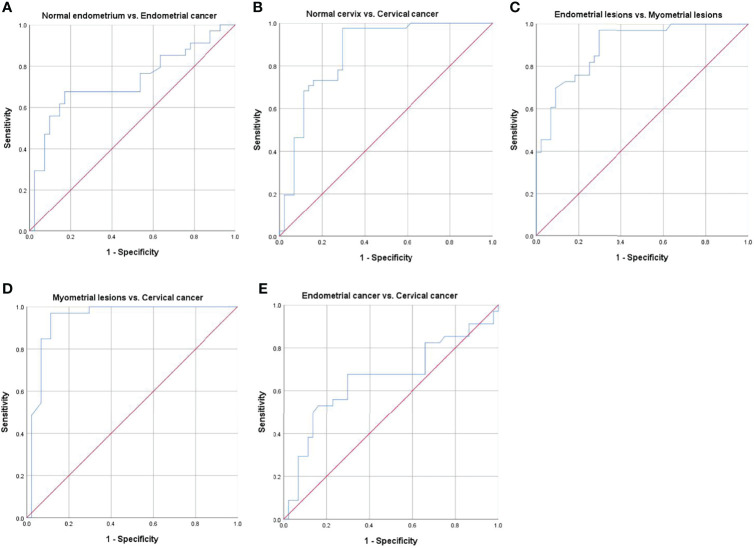
Receiver operating characteristic (ROC) curves for differentiating different groups. **(A)** normal endometrium vs. endometrial cancer, **(B)** normal cervix vs. cervical cancer, **(C)** endometrial lesions vs. myometrial lesions, **(D)** myometrial lesions vs. cervical cancer, and **(E)** endometrial cancer vs. cervical cancer.

Receiver operating characteristic curves and their related parameters were displayed in **Figure 5** and **Table 5**. Area under the curve (AUC), optimal threshold, sensitivity, and specificity for diagnosing endometrial cancer were 0.73, 7.90, 0.68, and 0.83, respectively. For diagnosing cervical cancer, they were 0.86, 7.94, 0.98, and 0.71, respectively. The AUC, optimal threshold, sensitivity, and specificity for differentiating myometrial lesions from endometrial lesions were 0.89, 8.95, 0.97, and 0.71, respectively. For differentiating myometrial lesions from cervical cancer, they were 0.94, 8.91, 0.97, and 0.89, respectively. For distinguishing endometrial cancer from cervical cancer, they were 0.66, 7.90, 0.68, and 0.71, respectively.

**Table 5:** Related parameters of receiver operating characteristic curves.

	AUC	95% CI	*P*	Threshold	Sensitivity	Specificity
Normal endometrium Endometrial cancer	0.73	0.61–0.85	0.001*	7.90	0.68	0.83
Normal cervix Cervical cancer	0.86	0.78–0.94	<0.001*	7.94	0.98	0.71
Endometrial lesions Myometrial lesions	0.89	0.82–0.96	<0.001*	8.95	0.97	0.71
Myometrial lesions Cervical cancer	0.94	0.89–1.00	<0.001*	8.91	0.97	0.89
Endometrial cancer Cervical cancer	0.66	0.53–0.79	0.02*	7.90	0.68	0.71

AUC, area under the curve; CI, confidence interval.

*, statistically significant difference with a value of P < 0.05.

As shown in **Table 6**, there was a critical positive correlation between age and MTR values in endometrial cancer (*r* = 0.81, *P* = 0.04). The correlations between age and the MTR values of other uterine lesions or normal uterine structures were not discovered (all *P* > 0.05).

**Table 6:** The correlations between age and the MTR values.

	Age (years)	MTR	*r*	*P*
Endometrial cancer (n = 34)	53.18 ± 11.09	8.29 ± 0.26	0.81	0.04*
Benign endometrial lesions (n = 10)	46.60 ± 7.18	7.99 ± 0.39	−0.58	0.08
Uterine leiomyoma (n = 25)	53.20 ± 10.18	10.54 ± 0.23	0.78	0.06
Uterine adenomyosis (n = 8)	53.13 ± 9.05	10.27 ± 0.47	−0.08	0.85
Cervical cancer (n = 44)	53.27 ± 10.26	7.71 ± 0.25	−0.06	0.71
Normal endometrium (n = 41)	52.22 ± 10.62	7.14 ± 0.21	0.08	0.61
Normal myometrium (n = 41)	52.22 ± 10.62	10.18 ± 0.22	0.05	0.75
Normal cervix (n = 41)	52.22 ± 10.62	9.51 ± 0.23	−0.08	0.63

MTR, magnetization transfer ratio.

*, P < 0.05.

## Discussion

In this study, we explored the value of MT imaging to characterize normal uterine structures and common uterine lesions by measuring MTR values. The results showed that the MTR values were significantly different among normal uterine structures, among uterine lesions of different origin, or between some uterine lesions and corresponding normal structures. MTR values were found to be effective in the diagnosis and differential diagnosis of certain uterine diseases. It might provide a preoperative basis for neoplastic histologic origin in the uterus.

Tissue contrast mechanism of conventional MRI is relying on density, T1 and T2 relaxation properties of free water protons, and diffusion properties of water molecules (19). It has a high sensitivity in detecting pathological tissue, but pathological specificity is poor (10). Except for leiomyoma and adenomyosis, almost all common uterine lesions show low signal intensity on T1WI and high signal intensity on T2WI (9). Malignant uterine tumors present high signal intensity on DWI due to high cell density and limited diffusion of water molecules (5, 9), whereas benign uterine tumors almost appear low signal (6, 9). DCE-MRI is associated with tumor vessel permeability and microvessel density (20, 21). Therefore, it is difficult to distinguish uterine cancers with poor blood supply by using conventional MR imaging alone. MT imaging can probe the protons bound to macromolecules and reflect the amount and complexity of immobile macromolecules in tissue and thus may have potential for providing excellent anatomical details and differentiating diverse pathological entities *in vivo* (13, 22). Measurement of MTR value may be more specific in detecting different structures (17). Kobayashi et al. (23) initially explored the application of MT imaging in cervical and endometrial tumors by comparing the signal intensity on multisection fast spin-echo (SE) images with that on a single-section fast SE image. The disadvantage of this approach was that the evaluation of MTR was limited to a single imaging section, and MTR values needed to be manually calculated after measuring on SE images. In this study, Gaussian RF pulse sequence was used to acquire MT imaging with shorter scanning time, and the MTR values could be measured directly on the MTR maps. Furthermore, the value of MT imaging in normal uterine structures was evaluated, and the correlations between age and the MTR values of the different uterine structures or different uterine lesions were explored. In our study, the MTR values showed significant differences among different structures of normal uterus. The tissue compositions of normal endometrium, myometrium, and cervix are various. The normal myometrium is composed of smooth muscle and fibrous connective tissue (24). The smooth muscle and fibration will increase the MTR values (17). The normal cervix consists of muscularis, stroma, and mucosa but contains only 10%–15% smooth muscle cells in cervical tissue (25). Therefore, the MTR values of normal cervix were lower than those of normal myometrium. The normal endometrium is made up of epithelial cells and lamina propria, lacking smooth muscle and fiber (26), which leads to the lowest MTR values.

MT imaging parameter might be an indicator of reflecting tissue integrity (16). This study found the significant differences between endometrial cancer and normal endometrium and between cervical cancer and normal cervix, which was consistent with the previous study (23). The invasive growth of cervical cancer would inevitably lead to destruction of normal cervical tissue, lead to decreased cervical fibrostroma and smooth muscle content, and then reduce the macromolecular substance content, potentially leading to lower MTR values of cervical cancer than those of normal cervix. Moreover, the MTR values of cervical cancer after radiotherapy would decrease, owing to tissue edema (23). However, we found that the MTR values of endometrial cancer were significantly higher than that of normal endometrium. One possible reason is that the proliferative growth of endometrial cancer would result in increased cellular density. An increase in the amount of tumor cells would lead to an increase in the cell membrane, and the content of macromolecules in the cell membrane would increase, thus potentially leading to increased MTR values of endometrial cancer. On the other hand, the aggressive growth of tumors would lead to changes of metabolic substances (27). Those metabolites included immobile macromolecular substances and mobile proteins and peptides (14). Endometrial cancer cells were more metabolically active than normal endometrial cells, potentially resulting in higher MTR values. The MTR values of endometrial cancer were significantly higher than those of cervical cancer in this study. The possible cause is the differences in histological types. Endometrioid adenocarcinoma is the most common subtype of endometrial cancer, and cervical cancer is mainly squamous cell carcinoma. Adenocarcinoma originates from endometrial cells with abundant glandular structures and has the ability to secrete mucins (28), potentially leading to higher MTR values. A systematic review and meta-analysis (10) confirmed that the pooled sensitivity and specificity for MRI in predicting origin of indeterminate uterocervical cancers were 0.884 and 0.395, respectively. Of which, T2WI and DCE-MRI were the most popular sequences, and DWI sequence and apparent diffusion coefficient values were also valuable. This study discovered the sensitivity and specificity were 0.68 and 0.71, respectively, by using MTR values to distinguish endometrial cancer from cervical cancer. Although sensitivity was reduced, specificity was significantly improved. In consequence, MT imaging with the non-invasive molecular level may potentially provide supplementary information in detecting and distinguishing uterine cancers. Different from the study of Kobayashi et al. (23), no significant differences were found between the MTR values of endometrial cancer and those of the benign endometrial lesions in this study. The possible reason was that the benign endometrial lesions included four cases of endometrial atypical hyperplasia considered as precancerosis of endometrial cancer.

Garcia et al. (16) demonstrated the differences in MTR values between glioblastoma multiforme and meningioma, which depicted that MTR values had the potential for differentiating different tumor types. Our study also found the MTR values could differentiate myometrial lesions from endometrial or cervical lesions. Adenomyosis and leiomyoma are common benign uterine lesions originating from myometrium, which is rich in smooth muscle cells. Hence, myometrial lesions had significantly higher MTR values than endometrial or cervical lesions. Boss et al. (29) found that a leiomyoma exhibited high MTR values during whole-body MRI, and the incidental finding was in conformance with our results. In addition to smooth muscle cells, myometrial lesions such as uterine leiomyoma are also composed of a large amount of extracellular matrix with proteoglycan (24). The macromolecular proteoglycan composition can increase the MTR values. Although myometrial lesions are not often mistaken for endometrial or cervical lesions on conventional imaging (e.g., T2WI), challenges still exist. For instance, adenomyosis may appear hypointense on contrast-enhanced MRI similar to endometrial cancer, uterine leiomyoma may distort the normal uterine anatomy, and some endometrial cancer is isointense to the myometrium on T2WI (30). Our study suggested that MT imaging could help to overcome some pitfalls of conventional MRI by the molecular level. Our consequences also support the idea put forward by another researcher that imaging signatures may predict pathology (31). Munro et al. (32) detected treatment effect of GnRH analog drugs in patients with leiomyoma by MT imaging and DCE-MRI. They revealed that DCE-MRI was sensitive to the vascular changes thought to accompany successful GnRH analog treatment of leiomyoma. However, there was no apparent treatment effect by MT imaging, although baseline MTR was negatively associated with initial uterine and fibroid volume. Therefore, compared with other functional MRI imaging, MT imaging has some shortcomings and needed to be further explored.

A previous study suggested that, compared with MT imaging, amide proton transfer (APT) imaging could better reflect tumor biological behavior by detecting mobile proteins and peptides (14). Recently, Zhang et al. (33) found that the content of mobile protein of different structures of normal uterus was different by utilization of APT imaging. Another study found that APT MRI could provide molecular-scale information for distinguishing endometrial cancer from leiomyoma, adenomyosis, and normal uterine myometrium (34). They found that the AUC, sensitivity, and specificity for differentiating endometrial cancer from leiomyoma and adenomyosis were 0.87 and 0.85, 83.3% and 76.7%, and 83.3% and 81.6%, respectively. The AUC, sensitivity, and specificity were 0.89, 0.97, and 0.71, respectively, for MTR values to distinguish endometrial lesions from myometrial lesions in our study. Both imaging methods showed high identification performance, whereas the total imaging time of APT imaging was as long as 7 min 33 s. The total imaging time of MT imaging was 2 min 42 s in this study. Perhaps MT imaging will serve as a more applicable clinical approach in evaluating normal uterus and uterine lesions. However, to achieve this potential value, multicenter studies with a large sample size are required in the future.

This study had several limitations. First, as a preliminary study, the sample size was relatively small. In addition, other rare uterine tumors, such as uterine sarcoma, were not included in our study. Future large prospective studies with more uterine lesions are needed. In addition, the insufficient sample size makes it impossible for this study to further study cancer lesions, such as invasiveness and lymph node metastasis. We will continue to collect cases to prepare for the study of the histopathological characteristics of cancer lesions. Second, because of the limitation of anatomical details on MT imaging, this study only included normal myometrium, endometrium, and cervix and did not measure MTR value of fine uterine anatomy like junctional zone. The improvement of MT imaging quality needs to be further investigated. Third, to obtain pathology as a standard reference, the normal myometrium, endometrium, and cervix that we measured were not from normal volunteers but from patients with carcinoma *in situ*. We will include normal volunteers to verify our results in future studies. Fourth, B1 correction was not performed due to lack of B1 correction setting in the MT sequence of MRI scanner that we used. Uneven B1 field might lead to uneven image signal, though the images with poor quality such as motion artifacts were excluded in this study. Finally, single-slice evaluation might introduce sampling bias and not reflect the intralesion heterogeneity. On the basis of improving MT imaging quality, volumes of interest will be delineated in our future research.

In conclusion, MTR values could distinguish normal uterine anatomies including myometrium, endometrium, and cervix; diagnose and differentiate uterine cancer; and differentiate myometrial lesions from endometrial or cervical lesions. MT imaging may be a promising imaging technique for the assessment of normal uterine structure and uterine lesions by providing molecular-scale information. A next step improvement in MT imaging technology and validation at molecular level may help address current challenges.

## Data Availability Statement

The raw data supporting the conclusions of this article will be made available by the authors, without undue reservation.

## Ethics Statement

The studies involving human participants were reviewed and approved by Medical Ethics Committee of the First People’s Hospital of Yunnan Province. The patients/participants provided their written informed consent to participate in this study.

## Author Contributions

QB designed the study, performed the statistical analysis, and wrote the manuscript. QL and SW modified and optimized MT imaging scanning parameters. QL, JY, JYY, JD, and FD scanned MT imaging. QL collected patient data. QB and YW revised the manuscript. YZ guaranteed the integrity of the entire study. All authors approved the submitted version of the manuscript.

## Conflict of Interest

Authors YW and SW were employed by Siemens Healthineers.

The remaining authors declare that the research was conducted in the absence of any commercial or financial relationships that could be construed as a potential conflict of interest.

## Publisher’s Note

All claims expressed in this article are solely those of the authors and do not necessarily represent those of their affiliated organizations, or those of the publisher, the editors and the reviewers. Any product that may be evaluated in this article, or claim that may be made by its manufacturer, is not guaranteed or endorsed by the publisher.
